# Academic service-learning nursing partnerships in the Americas: a scoping review

**DOI:** 10.1186/s12912-021-00698-w

**Published:** 2021-09-23

**Authors:** Adelais Markaki, Ong-on Prajankett, Allison Shorten, Maria R. Shirey, Doreen C. Harper

**Affiliations:** 1 WHO Collaborating Center for International Nursing, School of Nursing, University of Alabama at Birmingham, 1720 2nd Avenue South, Birmingham, AL 35294-1210 USA; 2 The Royal Thai Army Nursing College, 317/6 Ratchawithi Road, Ratchathewi, 10400 Bangkok, Thailand

**Keywords:** Academic nursing, Partnerships, Community-based, Service-learning, Primary health care, Universal health, Americas

## Abstract

**Background:**

Academic service-learning nursing partnerships (ASLNPs) integrate instruction, reflection, and scholarship with tailored service through enriched learning experiences that teach civic responsibility and strengthen communities, while meeting academic nursing outcomes. *Objective*: This scoping review aimed to identify, appraise, and synthesize evidence of community focused ASLNPs that promote primary health care throughout the Americas region.

**Methods:**

A systematic search of PubMed, CINAHL, Scopus, Google Scholar, and LILACS English-language databases was performed in accordance with PRISMA guidelines. Full-text articles published since 2010 were reviewed using an inductive thematic approach stemming from the “Advancing Healthcare Transformation: a New Era for Academic Nursing Report” and the Pan American Health Organization “Strategic Directions for Nursing.”

**Results:**

A total of 51 articles were included with the vast majority 47 (92.1 %) representing North America. Structured, established relationships between an academic nursing institution or program and one or more community serving entities resulted in high levels of effectiveness and innovation across settings. Five themes emerged: (a) sustaining educational standards and processes - improving academic outcomes (25.5 %), (b) strengthening capacity for collaborative practice and interprofessional education (13.7 %), (c) preparing nurses of the future (11.8 %), (d) enhancing community services and outcomes (21.6 %), and (e) conceptualizing or implementing innovative academic nursing partnerships (27.4 %). A synthesis of conceptual frameworks and models revealed six focus areas: communities/populations (26.2 %), nursing (26.2 %), pedagogy (19 %), targeted outreach (14.3 %), interprofessional collaboration (11.9 %), and health determinants (9.5 %). A proliferation in US articles, triggered by nursing policy publications, was confirmed.

**Conclusions:**

ASLNPs serve as mechanisms for nurses and faculty to develop and lead change across a wide variety of community settings and healthcare systems, develop scholarship, as well as for students to apply the knowledge and skills learned. Given the lack of geographically broad evidence, successes and challenges across U.S. partnerships should be viewed cautiously. Nevertheless, ASLNPs can play a critical role towards meeting the goal of universal health access and coverage through partnering with the education sector. Further investigation of grey literature as well as Spanish and Portuguese language literature from Latin American and Caribbean countries is highly recommended.

**Supplementary Information:**

The online version contains supplementary material available at 10.1186/s12912-021-00698-w.

## Background

Over the last decade, rising demands for greater interface between academia and the ‘real world’ have accelerated transformation in higher education across disciplines. Academic service partnerships (ASPs) are established strategic relationships between educational and clinical practice settings that advance practice, education, innovation and research, leveraging the talents of both partners and thus, advancing mutual interests and priorities [1]. Nursing partnerships between an academic entity and a healthcare organization serve as a mechanism for: (a) nurses to develop and lead change across healthcare systems, (b) students to use the knowledge and skills learned, optimize their potential to full-scope practice, and enhance lifelong-learning [2]. Key attributes of these partnerships include collaboration, leadership support across both organizations, engagement in scholarly activities that address community-based needs, and frequent partner communication [3, 4]. In North America, and predominantly in the United States of America (USA), ASPs are abundant. Yet, there is limited knowledge of their prevalence and characteristics beyond the North American context, chiefly in Latin America and the Caribbean (LAC). With renewed interest in service learning, the purpose of this paper was to identify academic service-learning nursing partnerships (ASLNPs) in the Americas region, describe their characteristics, main enablers and barriers, as well as offer recommendations for further development.

### History and evolution

Jointly championed since 2012 by the American Association of Colleges of Nursing (AACN) and the American Organization of Nurse Executives (AONE), ASPs are known as organizational structures in support of system change and service-learning opportunities that advance healthcare transformation [4]. Following recommendations by the Institute of Medicine [5] and Beal [1], AACN and Manatt Health issued jointly in 2016 a seminal policy document, known as the *“New Era Report”* [6]. This report identified *“a path for achieving an enhanced partnership between academic nursing and academic health centers to advance integrated systems of health care, achieve improved health outcomes, and foster new models of innovation”* ([6] p.3).

Although the North American literature is replete with descriptions of ASP pre-requisites, benefits, types, and workforce development effects, objective evidence of success is limited [1]. A qualitative study by Dobalian et al. [7] of a multisite ASP in the U.S. Department of Veterans Affairs Nursing Academy Program (VANAP) offers one of the few data-based reports. When outcomes associated with ASPs are evident, these are generally limited in scope to the results of a program evaluation [8] or one sole dimension, such as financial performance [9]. A recent integrative review supports that while mutual ASP benefits include educational, research, financial, and human resource outcomes [10], not one comprehensive report captures all these dimensions together. In the only multi-lingual systematic review of the global literature, DeGeest and colleagues [9] concluded that a standardized and methodologically sound framework to evaluate ASPs was lacking. According to the same authors, critical evaluation of 114 articles showed that 85 % of ASPs were located in the USA, 7 % in Canada, 5 % in Australia, and 3 % in other countries (Taiwan, West Africa, United Kingdom, and Switzerland). These findings suggest that either other countries are not publishing about their ASPs or that *“the strategic use of ASPs is more embedded in U.S. settings and is in an incipient state in other countries”* ([9] p. 451). The authors also observed a subsequent proliferation in ASPs, most notably in the USA, following publication of policy papers relative to advancement of the nursing profession. Hence, there is evidence that ASPs may be more prominent in the more resourced countries across the globe and are triggered by policy paper publication.

### Importance of ASLNPs

Service–learning, a key focus for ASPs in English language literature, is a teaching and learning strategy that integrates meaningful community service with instruction and reflection [11, 12]. Goals of service-learning are multifaceted and include provision of opportunities to enrich student learning experiences, increase confidence in problem-solving, teach civic responsibility, and strengthen communities [11, 13]. Students learn through purposeful and immersive community-based experiences integrated into their curriculum, and as a mutual benefit, patient and community needs are met [12, 14, 15].

ASLNPs are often shaped by multiple stakeholder needs, and exist in many different forms, serving a wide range of functions in the community. Mutual benefit is a critical factor for effective and sustained ASLNPs [12, 15–17]. Therefore, strategies to sustain mutual interest must be evident throughout the life course of the organization to promote trust and effective communication, as well as to ensure that services reflect shared missions and goals [15, 18, 19]. To this direction, the Community Campus Partnership for Health [20], a nonprofit membership organization in North America established in 1997, has been promoting health equity and social justice through partnerships between communities and academic institutions. Recommendations include the establishment of guiding principles, high-quality methods, robust metrics, and collaborative experiences that reflect agency values and goals [15, 20].

Partnering with the education sector to respond to health system needs for universal health access and coverage is one of the main objectives adopted by the Pan American Health Organization (PAHO). Two documents, *Strategy on Human Resources for Universal Access to Health and Universal Health Coverage* [21], and *Strategic Directions for Nursing in the Region of the Americas* [22] adopt a series of strategies and resolutions across the themes of policy, leadership, regulation, research, practice, and education to achieve universal health access and coverage in the region. Towards this goal, partnerships can play a critical role. As a technical arm of PAHO, our WHO Collaborating Center was tasked to explore how ASLNPs are conceptualized and operationalized in literature stemming from the Americas region. Therefore, the objectives of this scoping review were to: (a) identify community-focused ASLNPs in the Americas across English-language literature; (b) describe their characteristics and scope; (c) identify main enablers and barriers; and (d) offer recommendations for the Americas region.

## Methods

Stemming from the above objectives, a scoping review was chosen as a preliminary assessment of potential size and scope of available English-language literature to map the volume, nature, and features in this given field. Similar to a systematic review, it provides analytical transparency and rigor, without attempting to sum up best evidence or formally appraise the quality of research methodology [23]. The scoping review presents an overview of a potentially diverse body of literature generating hypotheses rather than testing them [24]. We applied Arksey and O’Malley’s [23] five-stage iterative process: (a) identifying research questions; (b) identifying studies; (c) selecting studies; (d) charting data; and (e) collating, summarizing and reporting results. No ethical approval was required for this type of methodology. To operationalize ASLNP, we used the following adapted definition from Beal [1]: *a structured, established relationship where an academic nursing institution or program and one or more community serving entities agree to cooperate in order to advance primary health care outcomes, resulting in high levels of innovation and effectiveness.* Proof of formal partnership was operationalized as having any of the following: Memorandum of Understanding, signed contract, joint funding, measurable goals, or ongoing program evaluation.

### Search strategy

**Fig. 1:**
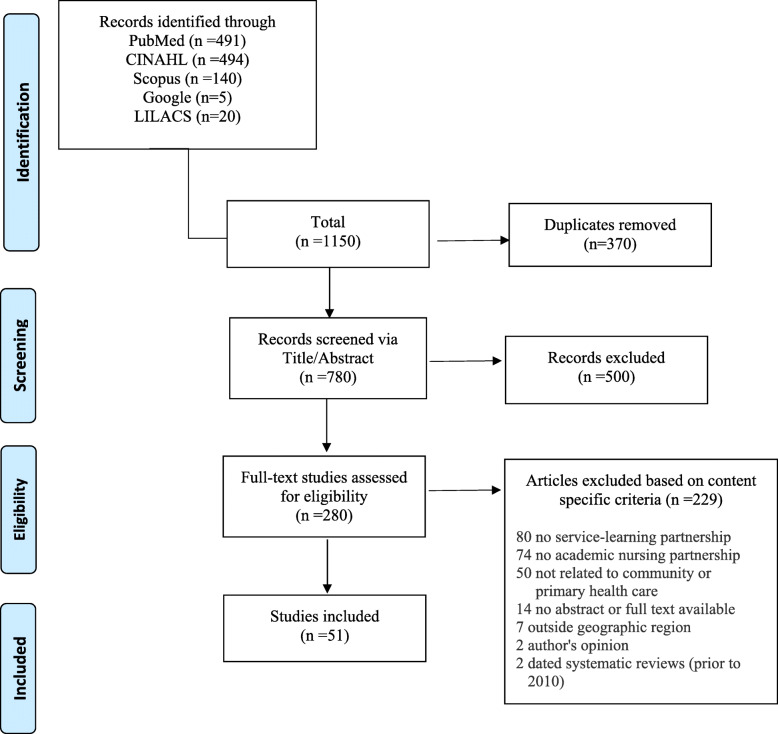
PRISMA search strategy

The Preferred Reporting Items for Systematic Reviews and Meta-Analyses (PRISMA) guidelines [25] were adhered to in the conduct and reporting of this scoping review. An electronic database search in PubMed, Scopus, CINAHL, Google Scholar, and Latin American & Caribbean Health Sciences Literature (LILACS) was performed using search terms that originated from indexed subject headings, the Medical Subject Headings (MeSH) terms, and keywords of relevant studies that recurred repetitively. The following terms, along with the Boolean operators ‘AND/OR’, were used: *“universities”, “academic”, “service”, “practice”, “community”, “health care”, “health-service”, “primary health care”, “public-private sector partnerships”, “partner”, “interinstitutional relations”, “schools, nursing”, “education, nursing”, “students, nursing”, “nurses.“* Electronic filters for full-text, English language, peer reviewed articles, published from 2010 to January 2020 were applied. A total of 1150 articles were retrieved and duplicates were removed with the use of Sciwheel Reference Manager. The resulting 780 articles were first screened by title/abstract. After excluding 500 articles that did not meet the inclusion criteria, 280 full-text articles were assessed for eligibility. During screening and eligibility steps, inclusion was determined based on meeting all five of the following content-specific criteria: (a) partnership between an academic nursing institution and another education institution or non-profit organization or professional organization or volunteer group; (b) partnership focuses on community-based or primary health care service/practice (including long-term care and home care); (c) partnership applies service-learning to teach or mentor or improve practice for nursing students or faculty of any program (undergraduate, graduate or professional); (d) partnership is described and/or measured through mutual goals or outcomes or deliverables or indicators; and (e) partnership within PAHO catchment region (North/Central/South America or Caribbean region). For a detailed search strategy, see Fig. 1 (PRISMA search strategy).

### Data extraction and quality appraisal

To attain validated scoping review processes, two independent investigators identified, selected, appraised, and synthesized all English-language literature. Each researcher independently screened title/abstract and full text and carried out data extraction and crosscheck. Through an inductive thematic analysis approach, the studies were first coded according to the main concepts addressed. Next, codes were grouped into sub-themes, which were eventually grouped into themes. Geographic distribution of the studies, along with the frequency and density of the themes, were analyzed. Any discrepancies throughout the above steps were resolved by a third investigator to reach consensus. Moreover, all selected articles were appraised for type and level of evidence according to the adapted *Rating System for the Hierarchy of Evidence* [26, 27]. In addition, evidence of ASLNPs was appraised and synthesized in terms of structure, process, and outcomes based on the Quality of Care approach [28]. According to this adapted approach, *structural* attributes were defined as the physical and organizational components of the setting in which the partnership occurred (i.e. nurse-led clinic). ASLNP *processes* were defined as learning methods, services or outreach provided to individuals, groups or communities (i.e. stroke risk assessment, health literacy). Last, ASLNP o*utcomes* were defined as the results of those processes (i.e. reduced emergency room visits, increased communication skills, staff retention rates).

## Results

A total of 51 articles was included in the final literature review sample, covering a 10-year period from January 2010-January 2020 (Supplementary File 1). Data were extracted for the following characteristics: author/s and year of publication, countries involved with ASLNP, study population/setting/sample, design, level of evidence, framework or model used, aim, type of ASLNP (formal, implicit or explicit), main findings (ASLNP structure, process, and outcomes), and implications.

Forty-six studies were conducted in the USA, two in Brazil [29, 30], one was a joint study between the USA and Haiti [31], another one between Canada and Colombia [32], and one study was conducted by USA investigators in Guatemala [33]. No English language studies were identified from other LAC countries. The majority of studies (76.5 %) involved multiple stakeholders (i.e. students, faculty, health professionals, preceptors, patients, community members), while 23.5 % focused exclusively on nursing students. Most of the studies were conducted in community-based organizations (29.4 %), some in public schools (17.6 %), free standing clinics (11.8 %), multiple settings (11.8 %), universities (9.8 %), VA medical centers (7.8 %), community hospital outpatient departments (5.9 %), public health departments (3.9 %), and one in home care (2.0 %). All studies involved a formal academic service-learning nursing partnership (Memorandum of Understanding, grant, contract or agreement) with 68.6 % of them demonstrating an implicit service-learning component, whereas only 31.4 % explicitly described the service-learning mission. Appraisal of type of study and level of evidence showed a prevalence of experiential and non-research evidence (51.0 %) at level VII, followed by descriptive/qualitative/mixed methods studies (41.2 %) at level VI, and in third place case-control or cohort studies (7.8 %) at level IV. A summary of evidence appraisal is presented in Table 1.

**Table 1:** Summary of evidence appraisal (*n* = 51)

**Countries with Academic Service-Learning Nursing Partnerships (ASLNP)**	**# of articles** (%)
USA	46 (90.1%)
Brazil	2 (3.9%)
USA and Haiti	1 (2%)
Canada and Colombia	1 (2%)
Guatemala	1 (2%)
**Study population**	**# of articles** (%)
Multiple disciplines/ stakeholders	39 (76.5%)
Nursing students	12 (23.5%)
**ASLNP setting**	**# of articles** (%)
Community-based organization or club	15 (29.4%)
School system (K-12)	9 (17.6%)
Free standing clinic	6 (11.8%)
Multiple settings^a^	6 (11.8%)
University setting^b^	5 (9.8%)
VA Medical Center	4 (7.8%)
Outpatient Clinic	3 (5.9%)
Public Health Department	2 (3.9%)
Home-based care	1 (2%)
**Type of ASLNP**	**# of articles** (%)
Formal [MOUs, contract, grant, agreement]	51 (100%)
Implicit service-learning	35 (68.6%)
Explicit service-learning	16 (31.4%)
**Level of evidence/ Type of study**	**# of articles** (%)
Level VII/ Experiential and non-research evidence	26 (51%)
Level VI/ Descriptive or qualitative or mixed methods study	21 (41.2%)
Level IV/ Case-control or cohort study	4 (7.8%)

^a^University, College of Nursing, Hospital, National Safety Council, Non-Governmental Organization, Residential Juvenile Justice Service, State Health Department, Community-based organization, Homeless Shelter ^b^Student Health Center, Wellness Center, School of Health Professions

**Fig. 2:**
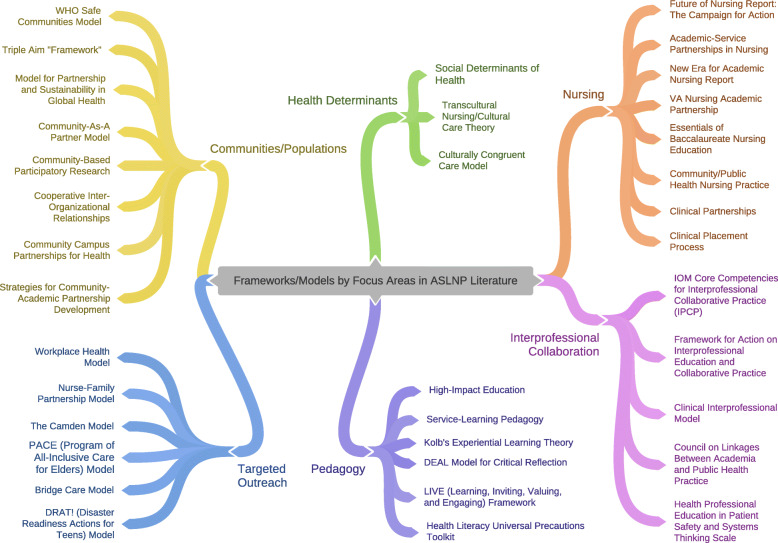
Mapping of frameworks/models by focus area

Furthermore, the extracted frameworks and models used by the sampled articles are presented in Table 2. Out of 51 selected articles, nine articles did not mention use of a framework or model. Among the remaining 42 articles, Service-learning Pedagogy, The Future of Nursing report, the VANAP Logic Model, Cooperative Inter-Organizational Relationships, Community-As-A Partner, and Transcultural Nursing/Cultural Care Theory were the most frequently adopted frameworks or models. Synthesis of these conceptual frameworks and models revealed the following six focus areas, mapped in Fig. 2: (a) communities/populations (26.2 %); (b) nursing (26.2 %); (c) pedagogy (19 %); (d) targeted outreach (14.3 %); (e) IP collaboration (11.9 %); and (f) health determinants (9.5 %).

**Table 2:** Meta-synthesis of frameworks/models in ASLNP literature by focus area (*n* = 42)^a^

Framework or model used in ASLNP literature	Citing article [Ref]	# of articles	Focus Area
Culturally congruent care model	[31]	1	Health determinants (*n* = 4)
Transcultural nursing/ Cultural care theory	[33, 34]	2	
Social determinants of health	[35, 36]	1	
Cooperative Inter-organizational Relationships	[29, 30]	2	Communities/ populations (*n* = 11)
Model for partnership and sustainability in Global Health	[31]	1	
Community-As-A Partner Model	[37, 38]	2	
Community Campus Partnerships for Health	[39]	1	
WHO Safe Communities Model	[40]	1	
Community-based participatory research	[41]	1	
Strategies for community-academic partnership development	[42]	1	
Community-based collaborative action research (CBCAR)	[43]	1	
Triple Aim (Institute for Healthcare Improvement)	[36]	1	
Clinical Placement Process	[44]	1	Nursing (*n* = 11)
The Future of Nursing (FON) report: the Campaign for Action	[45, 46]	2	
Essentials of baccalaureate nursing education	[47]	1	
Academic–service partnerships in nursing	[48]	1	
Community/public health nursing (C/PHN) practice	[49]	1	
VA Nursing Academic Partnership (VANAP) Logic Model	[50, 51]	2	
New Era for Academic Nursing (New Era Report)	[52]	1	
Essentials of baccalaureate nursing education for entry-level community public health	[53]	1	
Clinical partnerships	[54]	1	
IOM Core Competencies for Interprofessional Collaborative Practice (IPCP)	[55]	1	Interprofessional collaboration (*n* = 5)
Framework for Action on Interprofessional Education & Collaborative Practice	[56]	1	
Clinical Interprofessional Model	[57]	1	
Health Professional Education in Patient Safety and Systems Thinking Scale	[58]	1	
Council on linkages between academia and public health practice	[59]	1	
Service-learning pedagogy	[32, 60, 61]	1	Pedagogy (*n* = 8)
Kolb’s experiential learning theory	[33]	3	
High-impact education	[62]	1	
The DEAL Model for Critical Reflection	[38]	1	
LIVE (learning, inviting, valuing, and engaging) framework	[63]	1	
Health literacy universal precautions toolkit	[64]	1	
The Camden Model	[65]	1	Targeted outreach (*n* = 6)
Workplace Health Model	[66]	1	
Nurse-Family Partnership Model	[67]	1	
DRAT! Disaster readiness actions for teens	[68]	1	
Program of All-Inclusive Care for Elders (PACE) model	[69]	1	
Bridge Care Model	[70]	1	

^a^Out of 51 selected articles, 9 articles did not mention use of a framework or model while some used more than one framework/model

**Fig. 3:**
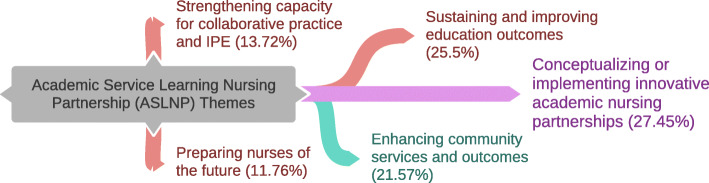
Mapping ofASLNP themes

**Fig. 4:**
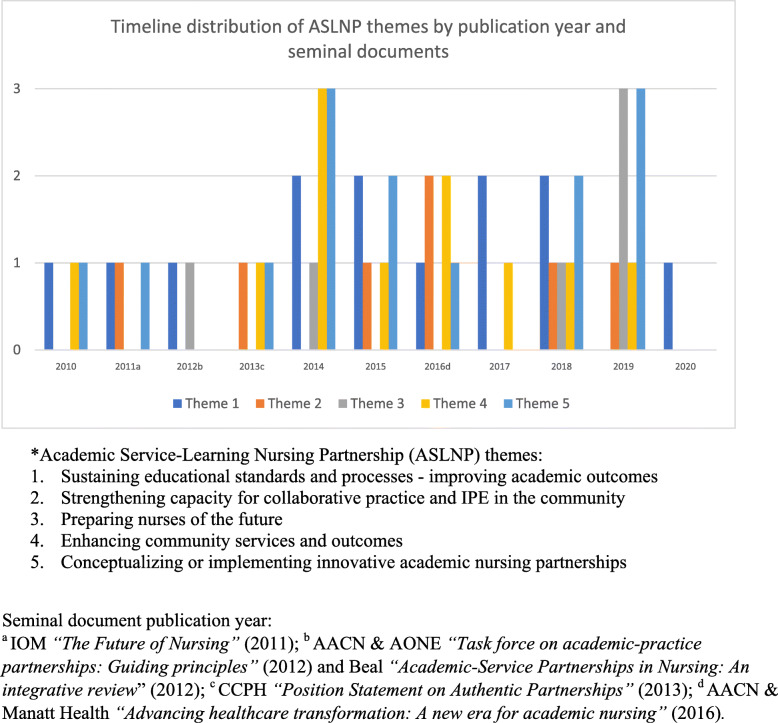
Timeline distribution of ASLNP themes by publication year and seminal documents. ^a^ IOM *“The Future of Nursing” *(2011); ^b^ AACN & AONE *“Task force on academic-practice partnerships: Guiding principles”* (2012) and Beal *“Academic-Service Partnerships in Nursing: An integrative review*” (2012); ^c^ CCPH*“Position Statement on Authentic Partnerships” *(2013);^d^ AACN & Manatt Health *“Advancing healthcare transformation: A new era for academic nursing” *(2016)

Last, synthesis of evidence revealed five ASLNP themes, depicted in Fig. 3. *Conceptualizing or implementing innovative academic nursing partnerships* was the most frequent ASLNP theme (27.45 %), followed by *sustaining educational standards and processes - improving academic outcomes* (25.5 %), *enhancing community services and outcomes* (21.57 %), *strengthening capacity for collaborative practice and interprofessional education (IPE) in the community* (21.57 %) and *preparing nurses of the future* (11.76 %). The timeline distribution of those five themes by publication year, in relation to the following seminal publications, is presented in Fig. 4: (a) Institute of Medicine *“The future of Nursing”* in 2011 [5]; (b) AACN & AONE *“Task force on academic-practice partnerships: Guiding principles”* [4] and Beal *“Academic-service partnerships in Nursing: An integrative review*” in 2012 [1]; (c) Community Campus Partnership for Health *“Position statement on authentic partnerships”* in 2013 [20]; and (d) AACN & Manatt Health *“New Era Report”* in 2016 [6].

Main findings addressing the nature, extent, and range of community focused ASLNPs that promote primary health care in the Americas region are highlighted below, grouped by themes.

### Theme #1: Sustaining and improving educational outcomes

A total of 13 articles focused on educational quality [30, 37, 39, 44–50, 62, 71, 72]. The structure of these ASLNPs consisted of schools of nursing, municipal health departments, outpatient clinics and health care networks, school districts, community clubs, and local community agencies. Within the unique structure of VANAP, this strategic alignment of academic and practice goals and resources targeted raising the capacity and capability of students, faculty and staff by providing health services to veterans [50].

Processes that sustained and improved educational outcomes involved: faculty practice models, faculty and clinical staff serving as preceptors, exchanging faculty and nurse practitioners, conducting health screenings, providing community-based education to elementary students, comprehensive health needs assessments, building trust, and making a long-term commitment. Aquadro et al. [45] found that the faculty practice model not only acknowledged the value of practice to academia, but also promoted and drove forward student and faculty teaching, learning and scholarship. Success of this model was attributed to being a recognized structure by the university, and allowing for diversity in professional practice and scholarship.

Several educational quality improvement outcomes were reported [30, 44–47, 50]: (a) decreased preceptor and faculty turnover rates; (b) continuous operation of nurse-managed clinics; (c) cost savings for training and on-boarding of new nurses; (d) sharing of nursing faculty expertise in research and clinical practice; (e) more relevant curriculum for students at all levels; and (f) integration of didactic knowledge and critical thinking skills among nursing students, while providing holistic nursing services. For example, in a study by Campbell et al. [47], students performed home safety and cognitive assessments while learning the challenges of nutritional needs of an adult population.

### Theme #2: Strengthening capacity for collaborative practice and IPE

A total of seven studies enhanced capacity for collaboration and IPE in the community [52, 55–57, 60, 65, 73]. These ASLNPs comprised of colleges of nursing, university health systems, affiliated hospitals, homeless shelters, VA Patient Aligned Care Teams, community health agencies, and public-school systems. Collaborative practice processes involved team-based care, partnering IP student teams with VA health professionals, preparing students to collaborate with local partners, providing care for vulnerable, underserved, high-risk, ethnically diverse adolescents. Based on the Institute of Medicine core competencies for IP collaborative practice, a faculty practice with residential juvenile justice services demonstrated how faculty and students served as leaders and experts in care coordination among ancillary providers [55]. Similarly, by partnering student IP teams with VA health professionals the students’ ability to link theory content to care delivery strengthened, resulting in better understanding of veteran population needs [57]. Other outcomes included [56, 65]: (a) learning and bonding together as an IP team; (b) preventing costly hospital readmissions; and (c) increasing awareness about social determinants of health. For example, the Interprofessional Community-Academic Navigation program facilitated homeless clients to access services while raising student awareness of the unique needs of this vulnerable population [65].

### Theme #3: Preparing nurses of the future

A total of six articles focused on preparing nurses of the future through partnerships with colleges of nursing, non-governmental organizations, a local catholic services agency, and an international service-learning nurse-led clinic [31, 33, 38, 58, 61, 66]. Processes involved global service learning, a train-the-trainer approach, provision of primary care by students and faculty, and student volunteer involvement in health promotion and needs assessment [31, 33, 38]. Through a global health immersion course that included an international nurse-led clinic, doctoral nursing students improved not only their clinical diagnostic skills, but also their cultural competence [33]. A study by Eustace et al. [38] applied the train-the-trainer approach using reflections and student peer evaluation to support development of global health competencies. Reported outcomes included: (1) increased student’s confidence in diagnostic abilities, (2) improved public health emergency competencies, (3) caring for a culturally diverse population, (4) understanding the impact of homelessness, and (5) learning how to use citizenship skills. As part of a leadership program that promoted competence in patient safety, pre-licensure graduate students were assigned to QI projects and were mentored by clinicians and faculty, preparing to become the future QI nurse leaders [58]. Benefits of this service-learning program extended to clinicians and faculty as well.

### Theme #4: Enhancing community services and outcomes

A total of 11 articles enhanced community health service access, and outcomes [32, 35, 40–42, 63, 67, 74–77]. Partnerships were comprised of schools of nursing, urban private universities, local health departments, elementary schools, local organizations, mobile clinics, and national organizations such as the National Association of Hispanic Nurses. Reported processes involved response to actual community health concerns, developing a home visit program, and building family-student partnerships. Main outcomes achieved were: (1) healthy lifestyle promotion, disease prevention and management by patient and family [41, 63, 75–77], (2) community support for safe medication disposal [40], (3) professional growth and development [63], (4) community engagement [76], and (5) access to care [32]. For example, one innovative partnership addressed illegal drug use in a rural setting by developing a sustainable drug education curriculum that was incorporated within an elementary public school [41].

### Theme #5: Conceptualizing or implementing innovative academic nursing partnerships

A total of 14 studies conceptualized a new or existing model or framework for ASLNPs [15, 29, 34, 36, 43, 51, 53, 54, 59, 64, 68–70, 78]. Partners included the office of health promotion and wellness, the National Program of Reorientation of Professional Formation in Health (Brazil), departments of public health, public schools, community rehabilitation facilities, community stakeholders, VA medical centers, academic nurse-managed clinics, and primary care clinics.

ASLNP processes involved implementation of the National Curriculum Guidelines in health science courses in Brazil [29], group activities, teaching health literacy, improved access to health service, and community collaboration to address social determinants of health. Within a public-school setting in the USA, the “Bridge Care Model” was used to access medical navigation support for vulnerable students and uninsured families [70]. Similarly, a 10-year partnership between the “Living Independently for Elders” members and a school of nursing in the USA, established community-based long-term care for high-risk older adults [69]. As part of VANAP, an endowed scholarship for students committed to caring for veterans and an after-hours clinic in a VA Medical Center were reported outcomes [51]. Other reported outcomes were: (1) a positive perception of ASLNP [29], (2) a model for delivering community health instruction [53], (3) increased cost-effectiveness in terms of school nurse manpower [68], (4) reduced delays in school enrollment [70], (5) ability to translate research evidence into practice [51, 64], and (6) increased engagement with policy makers at the state and national level [69].

## Discussion

This scoping review provided a preliminary assessment of the potential size and scope of available English-language literature and identified the nature and extent of research evidence on ASLNPs within the PAHO region. Of note, only two articles [29, 30] originated from LAC countries. With a constant focus on our objectives and the inherent limitations, we discuss the main characteristics and scope of ASLNPs, enablers and barriers, identified similarities and differences, and offer some recommendations.

### Characteristics and scope

All articles described a formal partnership with presence of an MOU, grant, contract or agreement. One third of those partnerships had an explicit service-learning mission, whereas two thirds demonstrated implicit service-learning components. Multiple disciplines/stakeholders, including nursing students and faculty, comprised the study populations, with most of the studies taking place in community-based organizations, public schools, free standing clinics, universities, VA medical centers, community hospital outpatient departments, and public health departments. The vast majority of articles provided descriptive/qualitative/mixed methods evidence or experiential and non-research evidence. Academic nursing partnerships with community, federal, and non-profit organizations in North America have attempted to bridge gaps in PHC and population health services by building on the service-learning component. Among the sampled literature, only one article reported an ASLNP in affiliation with a WHOCC in Community Safety Promotion to prevent opioid abuse through a public health practicum [40]. Counter-intuitively, six other articles from US institutions with designated WHOCCs for Nursing and Midwifery did not appear to stem from a WHOCC-related activity. Those ASLNP initiatives focused on: teaching team-based care through a nurse-led clinic [56], preparing students for IP collaboration with local partners [60], establishing a long-term care community program for high-risk elderly [69], advancing patient safety competence along with student leadership [58], and improving quality of care for veterans [50, 51]. Such partnerships have been shown to be further enhanced by the use of an IP collaborative practice model for chronic disease management coordination [79]. Hence, all evidence points to the link between successful leveraging of resources among partners and advancing health with underserved populations.

Synthesis of conceptual frameworks and models, used by the sampled articles, showed the following focus areas: communities/populations and nursing, followed by pedagogy, targeted outreach, IP collaboration, and health determinants. The small number of articles focusing on IP collaboration and health determinants is not surprising given the academic disciplinary approach that has been prominent in the past. This trend seems to be changing over the last 2–3 years, with a more accelerated pace expected in the U.S. literature brought about by the COVID-19 pandemic and the widespread movement to address social justice and healthcare inequalities. Given the convergence of these urgent demands on healthcare systems and nursing’s frontline role across the Americas, new focus areas may potentially evolve in ASLNPs.

The scope of ASLNPs, illustrated in Fig. 3, was captured through five emerging themes: (1) *sustaining educational standards and processes - improving academic outcomes* (i.e. clinical placement, preceptorship, quality improvement); (2) *strengthening capacity for collaborative practice and IPE in the community;* (3) *preparing nurses of the future* (recruitment, mentorship, job transition, mobility, civic responsibility, either faculty or student); (4) *enhancing community services and outcomes* (extended hours, access to PHC services, wide scope of services, universal coverage); and (5) *conceptualizing or implementing innovative academic nursing partnerships* (agenda, policy, funding, frameworks/models). Not surprisingly, the timeline distribution of those themes in relation to seminal document publication year (Fig. 4) showed that theme #3 has been more prevalent since 2018, whereas themes #4 and #5 peaked in 2014, following publication of seminal policy documents. Hence, the earlier observation by DeGeest et al. [9] of a proliferation in U.S. articles, triggered by nursing policy paper publication, is confirmed.

### Enablers and barriers

Four enablers for strong ASLNPs were identified: (1) guiding principles, (2) quality processes, (3) meaningful outcomes, and (4) transformative experiences. All were consistent with those reported among seasoned community and academic partners engaged in authentic partnerships [20]. However, for ASLNPs with a specific focus on collaborative practice and IPE (theme #2), an array of inherent barriers to IPE were reported in relation to: curriculum, leadership, resources, stereotypes and attitudes, variety of students, teaching, and enthusiasm. In agreement with a 2014 systematic review across developing and developed countries [80], the presence of flexible, enthusiastic institutional champions and resources were key determinants of success in embedding meaningful IPE learning opportunities as part of a health professions core curriculum and overcoming administrative barriers. Faculty and workforce development were also an essential component for successful ASLNPs that promote IPE and IP practice [30, 32, 45, 46, 50, 51, 54–56, 60, 73]. In addition to investment in educational technology, partnerships enabling faculty practice to build expertise and spark the cultural change were essential for overcoming the pervasive stereotypes, skepticism, and long-held attitudes about other professions. Most of these ASLNPs were developed individually, rather than systematically, through established public health or governmental programs. Similarly, many were started from grants and/or philanthropic and in-kind donations based on individual relationships which contributes to sustainability challenges. The need for further research to identify best-practice models for integrating IPE as core curriculum, communicating consistent expectations for ASLNP outcomes, and seeking perspectives of patients and community partners regarding their experiences in partnership with learners has been underscored [15]. Being aware of these challenges and barriers in advance, academic nursing institutions in LAC countries will be more prepared and can enhance the partnerships’ potential success.

### Similarities and differences

Despite recognized challenges and barriers in establishing and sustaining ASLNPs, evidence points to numerous benefits including fostering collaborative practice and IPE, improving professional satisfaction, and ultimately improving patient care and outcomes. However, the majority of evidence comes from North America, with only five articles from LAC countries. Additional analysis of the literature sample showed consistencies across service-learning structures, processes and outcomes. ASLNPs provide enhanced clinical experiences for nursing students, targeted population health for vulnerable or marginalized groups, student/faculty engagement in community awareness and well-being, and cultural/global health competency development. Twenty-one articles from the USA demonstrated integration of public health perspectives/needs with nursing program accreditation standards, and student leadership enhancement. Fourteen articles focused on health promotion and disease prevention, especially in the school setting. Eleven articles examined development of faculty practice, clinical skills, and preceptorship through innovative partnerships for clinical placement. Among those innovative partnerships, VANAP allowed for transformation of veterans’ health care through educational, research, and clinical practice outcomes achieved by students, practitioners, and faculty [50, 51]. Designed to address the nursing workforce shortage, the VA framework contextualizes the local needs and demands of all partners [7]. Recipients of VA services have access to an array of essential services comparable to universal health coverage. In comparison, articles from Brazil [29, 30], Haiti [31], Guatemala [33], Canada and Colombia [32] focused on the engagement of nursing faculty and students with culturally diverse, underserved populations and the acquisition of cultural competence skills gained while performing community diagnostics, home visits, and health education.

Beyond the value to partnering practice organizations, the benefits of ASLNPs for students included greater confidence in problem solving, and development of professional competence. Learners were able to build relationships with patients and community agencies, some of them longer term, and gain insight into unique needs as well as strengths of the community and its members. There was also evidence that students grew personally in their understanding of other cultures, some gaining cultural competencies in an international context, through collaboration with other professions and engagement in multi-disciplinary teamwork. These benefits are consistent with those previously reported [13]. Likewise, the benefits of ASLNPs for faculty included opportunities to engage in social justice and real-world scholarly activities that build grass-roots community-based capacity. Yet, there was no evidence of the effect of structural or systemic inequalities on ASLNPs, despite the emerged importance of cultural competence and safety in *Preparing Nurses of the Future*. These experiences expand academic nursing’s contributions beyond traditional acute care settings, broadening nursing’s social impact.

### Recommendations for nursing practice and policy

This scoping review was based primarily on North American literature and academic-service policy statements. Given the lack of geographically broad evidence, adoption of ASLNPs in LAC countries based on assumptions and tools derived mostly from the US experience may need modification. According to the two Brazilian studies [29, 30], the authors describe a “cooperative inter-organizational” relationship that integrates education and service in PHC. Clearly this model differs from the ones adopted by investigators of North American studies. Hence, successes and challenges encountered across USA academic nursing partnerships focusing on service-learning, might be culturally biased and therefore, one should proceed cautiously with a broad plan of action for LAC countries.

Developing a joint policy paper with representation from key stakeholders in the Americas, while adapting the AACN toolkit, could facilitate ASLNP proliferation throughout the region. According to the New Era Report [6], partner representatives from each side of academia and service should have “a seat at the table” to plan the advancement of ASLNPs that address joint needs and objectives. Considering the ability of each partner to leverage joint resources for advancing a shared agenda is critical. However, the cultural differences in nursing education across the Americas predicate that a sequence scoping review of multi-lingual studies be conducted, before these frameworks are generalized. It is indeed plausible that academic-service community-based partnerships in LAC countries are already integrated in nursing education programs; therefore, they are not viewed as novel or unique. According to Santos [81], the theoretical frameworks of the global South differ greatly from those of Western societies (the global North). Acknowledging the different ways of knowing, by which people across the global South live their lives and provide meaning to their existence, is an important step as we explore ASLNPs in the future. Building upon diverse sources of knowledge and experience in nursing is an important step towards achieving global social justice in health.

Following the release of the *“State of the World’s Nursing − 2020”* report [82], and the rapidly deployed changes as a result of the COVID-19 pandemic, optimizing and re-evaluating ASLNPs is highly recommended. The National Academy of Medicine’s [83] vision and path for the nursing profession to create a culture of health, reduce health disparities, and improve the health and well-being of the USA population in the 21st century could offer a starting point. Countries where nursing has a seat at the national health policy table should partner with institutions from less-privileged countries to build the evidence from different geographical, cultural and political contexts. The previously identified need for a stepwise model to accommodate countries of varying academic nursing capacity and resources within the same region should be considered [84]. Future reviews should include the grey literature on the topic (i.e. government reports, policy papers, academic documents, etc.), while research studies should address literature gaps through empirical inquiries. A more comprehensive understanding of expected outcomes for service-learners and recipients of services, as well as ASLNP challenges in countries with different educational and healthcare systems will facilitate next steps.

### Strengths and Limitations

This review was based on an extensive search of five electronic databases spanning ten years. Special emphasis was given to resources from Latin America and the Caribbean by searching through the LILACS database, a comprehensive index of scientific and technical literature from this region. All articles were independently reviewed, and level of evidence was appraised with an adapted hierarchy scale [26, 27], classifying the vast majority as level VI or VII which indicates a low level of evidence. However, it is important to note the ongoing debate over the applicability of the hierarchy of evidence when evaluating qualitative nursing research [85].

Furthermore, we only searched for English-language peer-reviewed publications, which would have excluded any relevant grey literature from the targeted region published in Spanish or Portuguese languages. the grey literature on the topic (i.e. government reports, policy papers, academic documents, etc.) should be scanned as well in order to get a comprehensive picture. As the search yielded only five articles from the LAC region, we first reviewed the 46 articles from the North American region. Then, after identifying common challenges and barriers, we compared them to the five articles from LAC countries to develop recommendations for the PAHO region. The outcomes of interest, barriers and challenges, were mostly measured qualitatively which was consistent with the qualitative nature of our aim. Other potential limitations included selection bias and variation in criteria application, upon appraisal. Last, cross-country differences in the education and health system, such as regulation and accreditation of nursing programs, existence of a national health system, as well as cultural and linguistic variations are acknowledged.

## Conclusions

This scoping review illustrated that ASLNPs are more prominent in the U.S. literature, rather than in literature from LAC countries, and seem to be detected when nursing policy papers are published. The expansion of nursing’s role in the community, coupled with increased focus on primary health care, public health, and population health capacity in the nursing workforce in North America are contributing factors to the development and growth of these strategic partnerships. The embrace of these partnerships across the Americas, drawing on the strengths and challenges of each country, and their impact on education and practice environments of future nurses remains to be further explored. Collaboration for ASLNP knowledge development and integration will strengthen and advance academic nursing education in response to PAHO’s call for universal health access and coverage across the Americas.

## Supplementary Information

**Additional file 1.** Literature review data extraction and appraisal.

## Data Availability

All data generated or analyzed during this study are included in this published article.

## Abbreviations

AACN: American Association of Colleges of Nursing
AONE: American Organization of Nurse Executives
ASLNP: Academic Service-Learning Nursing Partnership
ASP: Academic Service Partnership
IP: Interprofessional
IPE: Interprofessional Education
LAC: Latin American and Caribbean
PAHO: Pan American Health Organization
PHC: Primary Health Care
QI: Quality Improvement
VANAP: Veterans Affairs Nursing Academy Program
WHO: World Health Organization
