# Supplementary material for: Academic service-learning nursing partnerships in the Americas: a scoping review
Source: BMC Nurs. 2021 Sep 23;20:179. doi: 10.1186/s12912-021-00698-w (PMC8459147; doi:10.1186/s12912-021-00698-w)
Supplement: Supplementary file 1 — Additional file 1. Literature review data extraction and appraisal. [file 12912_2021_698_MOESM1_ESM.docx]

**Supplementary File 1:** Literature review data extraction and appraisal – academic service-learning nursing partnerships (ASLNPs) (n=51)

| **Author(s), year,**  **countries included** | **Study population/**  **Setting/**  **Sample** | **Design/**  **Level of evidence*** | **Framework or model used (source)** | **Aim /Type of ASLP (formal/**  **explicit or implicit)** | **Findings (ASLNP structure, process, outcomes)** /**  **Implications** | **ASLNP Theme §** |
| --- | --- | --- | --- | --- | --- | --- |
| Alexander et al., 2014 [40]  USA | Urban private university & Safe Communities America, a program of the National Safety Council in affiliation with WHOCC in Community  Safety Promotion/  n=10 nursing students | Descriptive narrative/  Level VI | “WHO Safe Communities Model”  (National Safety Council, 2014) | Population-focused public health nursing practicum to promote prevention of opioid abuse  Formal/Explicit | *Structure:* student task force to promote prevention of opioid abuse  *Process:* injury prevention/opioid overuse  “take back meds” and “meds drop box” initiatives  *Outcomes:* 1) community support for safe medication disposal, 2) students demonstrated professional accountability to the community and multidisciplinary stakeholders.  *Implications:* ASLPs promote community engagement among students and facilitate the development of public health nursing projects. | #4 Enhancing community services and outcomes |
| Andrade et al., 2014 [29]  Brazil | Community-based organization /Students, faculty,  Pró-Saúde I Project leaders  (n=8) | Qualitative study /  Level VI | “Cooperative Inter-Organizational Relationships”  (Ring & Van de Ven, 1994) | Education-service integration, with the aim of redirecting nursing training toward PHC  Formal/Explicit | *Structure:* National Program of Reorientation of Professional Formation in Health (Pró-Saúde): a governmental cooperation initiative between health and education sectors  *Process:* implement the National Curriculum Guidelines (NCGs) in the UG courses of the health sciences.  *Outcomes:*  a positive perception of ASLP in regards to training and continuing education objectives.  *Implications*: future research should include student and user’s perceptions to interpret ASLP relationships. | #5  Conceptualizing or implementing innovative academic nursing partnerships |
| Andrade et al., 2015  [30]  Brazil | Municipal Health Departments & undergraduate (UG) nursing program /  n=16 UG nursing faculty & PHC preceptors | Qualitative study / semi-structured interviews / Level VI | “Cooperative Inter-Organizational Relationships”  (Ring & Van de Ven, 1994) | Established by contract between academic institution and municipal health departments for PHC preceptorship  Formal / Implicit | *Structure:* nursing clinical in PHC setting  *Process:* faculty and clinical staff as preceptors  *Outcomes:* identified obstacles included need for space, preceptor and faculty turnover rates.  *Implications:* further studies to focus on the inter-organizational relationship between university and healthcare units in the context of the learning process as well as the community served. | #1 Sustaining educational standards/ processes - improving academic outcomes |
| Aponte et al., 2010  [44]  USA | Urban college Wellness Center (WC) and nursing school /  nursing students & faculty  (n=40) | Descriptive  Narrative/ Level VI | “Clinical Placements Process”  (Massachusetts Board of Higher Education, 2009) | Collaborative intra-institutional partnership for clinical placements and preceptorship  Formal / Implicit | *Structure:* SON faculty and clinic staff served as preceptors  *Process:* nursing students conducted screenings, health promotion for college students  *Outcomes:* WC clinical site allowed students to assess, integrate, and synthesize didactic knowledge and critical thinking skills while providing holistic nursing services.  *Implications:* ongoing partnership bridged service and academic needs of both entities | #1 Sustaining educational standards/ processes - improving academic outcomes |
| Aquadro et al., 2014  [45]  USA | Local hospital / SON faculty practice – NPs/nursing students/nurse managed clinic  n=3 NPs  n= 4-6 students | Descriptive narrative/ Level VI | “The Future of Nursing (FON) report: the Campaign for Action” (IOM, 2011) | To provide a clinical practice site for faculty, clinical instruction for nursing students and services to an underserved population  Formal/Implicit | *Structure:* a regional university, city housing authority, local hospitals, tri-county dental association, United Way, and other community organizations.  *Process:* faculty practice model, clinical site instruction for UG nursing students and primary health care services to underserved  *Outcomes:* 1) patients received assistance, 2) SON and clinic representatives signed a mutual contract, 3) continuous operation of nurse-managed clinic with faculty *Implications:* enablers include a structure recognized by the university, allowing for diversity of professional practice and scholarship in the university. | #1 Sustaining educational standards/ processes - improving academic outcomes |
| Breen et al., 2019  [75]  USA | Public schools / 42 undergraduate nursing students | Qualitative study/  Level VI | N/A | Academic‐practice partnership to improve health outcomes for children with asthma  Formal/Implicit | *Structure:* Asthma coalition, public schools, SON  *Process:* population-focused clinical experiences for nursing programs and improving asthma self-management for children  *Outcome:* 1) student learning in positioning, professional identity, and social awareness, 2) data sets linking student learning and the goals, milieu and workings of the partnership.  *Implications:* community-based ASLP to improve population health outcomes offered unique clinical learning opportunities for students through exposure to values, ideas, and innovation. | #4 Enhancing community services and outcomes |
| Byrne et al.,  2014  [31]  USA/Haiti | Academic institution / non-governmental organization/ advanced practice nursing students (PCNPs) /  n= 17 students  n=4 faculty and n=3,000 patients | Program development & evaluation  through debriefing and focus groups/ Level VII | “Culturally congruent care model” (Schim et al., 2007)  and  “Conceptual model for partnership and  sustainability in Global Health^”^  (Leffers & Mitchell, 2011) | Academic partnership for global health and cultural competency  Formal/ Explicit | *Structure:* College of Nursing and a US nongovernment organization at 5 sites in Haiti  *Process:* NP students involved in global service learning (short-term study abroad)  *Outcomes:* students reported greater confidence in advanced practice and cultural competency skills  *Implications:* need for a formal evaluation tool of global health competencies for NP students. Competencies by Wilson et al^5^ may serve as a springboard. | #3  Preparing Nurses of the Future |
| Calvert et al., 2014  [41]  USA | Elementary school / Nursing students/ faculty/ community partner/ elementary students | Qualitative study focusing on planning, pilot testing, and evaluation / Level VI | “Community-based participatory research” (Israel et al., 1998) | Community partners and faculty collaborated to address meth use in a rural setting  Formal/Implicit | *Structure:* community partners and university faculty  *Process:* incorporated a sustainable drug education curriculum in an elementary school and provided learning opportunities for senior nursing students  *Outcomes:* community received early elementary school drug education, and nursing students were actively involved with actual community health concerns  *Implications:* implemented a free, science-based curriculum developed by the National Institute on Drug Abuse to explore drug effects on the brain. | # 4 Enhancing community services and outcomes |
| Campbell et al., 2018  [47]  USA | Home-based care/ Nursing students, senior adults, nursing faculty  n=120 students | Program development & evaluation (formative & summative) / Level VII | “Essentials of baccalaureate nursing education”  (American Association of Colleges of Nursing, 1998) | Collaborative care for homebound senior adults enrolled in home delivery meal services  Formal/Implicit | *Structure:* The Meals on Wheels (MOW) partnership, College of Nursing  *Process:* MOW program; students performed home safety and cognitive assessments while learning the challenges of nutritional needs of an adult population.  *Outcomes:* an innovative placement to meet nursing program outcomes and clinical practice hours.  *Implications:* areas for future growth include interdisciplinary collaboration and partnering with local hospitals to study impact on hospital readmission rates. | #1 Sustaining educational standards/ processes - improving academic outcomes |
| Carter et al.,  2013  [53]  USA | Office of Health Promotion and Wellness / nursing students and faculty, university employees /  n=320 nursing students | Cohort study / Level IV | “Essentials  of baccalaureate nursing education for entry-level community public health nursing^”^  (Association of Community Health Nurse Educators, 2010) | Collaborative approach for improving community health instruction  Formal/Implicit | *Structure:* College of Nursing and the Office of Health Promotion and Wellness  *Process:* Collaborative Model (WellBAMA); community health nursing students provide health promotion for university employees  *Outcomes:* Better NCLEX scores, better prepared for practice  *Implications:*  SON could partner with community health centers and senior citizen programs for health promotion clinical opportunities. | #5  Conceptualizing or implementing innovative academic nursing partnerships |
| Clifton et al., 2016  [55]  USA | Residential Juvenile Justice Services (JJS) facilities and College of Nursing/  Faculty  Nursing Students  JJS residents | Descriptive narrative/ Level VI | “IOM Core competencies for interprofessional collaborative practice (IPCP)^”^  (Interprofessional Collaborative Expert Panel, 2011) | Initiate faculty practice sites, education, research, and service  Formal/Implicit | *Structure:* Residential JJS facilities and College of Nursing  *Process:* provide care for vulnerable, underserved, high-risk, ethnically diverse adolescents  *Outcomes:* mutually beneficial for College of Nursing, students, faculty, and JJS  *Implications:* innovative faculty practice model that can be replicated throughout the country | #2 Strengthening capacity for collaborative practice and IPE in the community |
| Commenda dor et al., 2016  [74]  USA | Elementary school SON, elementary schools/  nursing students, children, community groups | Descriptive narrative/ Level VI | N/A | Involve local school children as health educators  Formal/Implicit | *Structure:* SON, elementary school, and local organizations  *Process:* Nursing students taught middle school students how to recognize high BP and prevent stroke  *Outcomes:* successful for preparing nurses, teaching prevention to elementary school students, and reducing stroke risk in the community.  *Implications:* this project initiated health promotion education in the community. | # 4 Enhancing community services and outcomes |
| Connor et al., 2010  [35]  USA | Mobile clinic / Migrant farm workers (n=700-1000),  Health profession students (n=90) | Community assessments,  Literature review/ Level VII | “Social determinants of health” (CDC, 2005) | Strengthen health programs that serve migrant farm workers  Formal/Implicit | *Structure:* SON, local partners, and mobile clinic  *Process:* Farm Worker Family Health Program (FWFHP) strategies  *Outcome:* nursing practice strategies address migrant farm worker’s health challenges  *Implications:* The FWFHP model may be adapted for low-resource setting populations | # 4 Enhancing community services and outcomes |
| Creech et al., 2018  [48]  USA | HIV/HCV clinics /  NP students,  Preceptors, HIV+ patients, their families and the LGBT community | Program development & evaluation/ Level VII | “Academic–service partnerships in nursing” (Beal, 2012) | Increase clinical placements for nurse practitioner students  Formal/Implicit | *Structure:* HIV/HCV clinics and health care networks, SON  *Process:* community assessment, trust-building, long-term commitment  *Outcome:* student readiness to practice and student satisfaction with clinics and preceptors  *Implications:* providing HIV clinics and community organization as placement sites increased the availability of clinical sites. | #1 Sustaining educational standards and processes |
| Cygan et al.,  2018  [52]  USA | Public school / Nursing students (n=79)  Public school students (n=2,000) | Program evaluation/ Level VII | “New Era for Academic Nursing report”  (AACN, 2016) | Partnerships to increase the school district’s capacity and nursing students’ competency  Formal/Implicit | *Structure:* public school system and a College of Nursing  *Process:* increase the school district’s capacity to meet sexual health education requirements while providing graduate nursing students with an opportunity to work with a diverse population of youth  *Outcome:* over 2,000 public school students received sexual health education, 79 nursing students were placed for public health nursing practicum and met clinical objectives  *Implications:* ASLPs are an important mechanism to simultaneously meet the growing needs of community practice partners and nursing education programs, while strengthening public health nursing practice | #2  Strengthening capacity for collaborative practice and IPE in the community |
| Dabney et al., 2017  [71]  USA | SON and elementary school district /  School nurses and elementary school students | Descriptive narrative/ Level VI | N/A | Enhance RN to BSN students’ clinical experiences at public schools.  Formal/Implicit | *Structure:* school district and SON  *Process:* provide PHC to elementary students  *Outcome*: partnership met the school’s needs and nursing student’s need for clinical hours  *Implications:* ASLP is mutually beneficial in terms of innovative clinical placements to higher education students and enhanced education to elementary students | #1 Sustaining educational standards and processes - improving academic outcomes |
| Davis et al.,  2015  [67]  USA | Community-based organization /  BSN students (n=53)  Families served (n=20) | Mixed methods /  Level VI | “Nurse-Family Partnership Model” (2013) | Promote healthy families and communities by creating academic-practice partnerships.  Formal/Implicit | *Structure:*  young vulnerable families and SON  *Process:* engaging BSN students in public health nursing (PHN) and building family-student partnerships supported by a “village” of interconnected resources  *Outcome:* Students reported linking theory with practice and valued the PHN practicum experience. Nine tools were developed including a GIS mapping strategy. Students provided 202 educational interventions, 39 community resource connections, and 46 care transitions.  *Implications:* ASLPs can guide students and families in a reciprocal relationship with resources. Home visiting can only be effective when the necessary infrastructure is in place. | #4 Enhancing community services and outcomes |
| Eustace et al., 2018  [38]  USA | Community-based organization /Senior nursing  students enrolled in community/  public health course who completed a “global” project (n=8) | Program development & evaluation/ Experiential and non-research evidence / Level VII | “Community-As-A Partner model” (Anderson & McFarlene, 2008)  and  “The DEAL Model for Critical Reflection” (Clayton et al. 2009) | To enable nursing students to: 1) demonstrate a better understanding of global health issues from a local perspective, 2) communicate effectively with people of diverse cultures, 3) understand responsibilities of global citizenship  Formal/Implicit | *Structure:* a local Catholic Services agency and SON  *Process:* train-the-trainer (TTT) approach was used to support student development of global health competencies and community capacity for working with refugees. A quiz, reflections, participant evaluations, and student peer evaluations were used for assessment of the learning experience.  *Outcome:* 1) TTT method prepared students in global migration and public health emergency competencies, 2) the project increased students’ awareness of local resources, including location and eligibility requirements.  *Implications:* teaching global health content requires an array of methods, including use of reliable tools and reflective practice. | #3  Preparing Nurses of the Future |
| Evans-Agnew et al., 2017  [49]  USA | Nursing students, SON,  3 hospitals,  1 State Health Department | Case study/ Experiential and non-research evidence / Level IV | “Community/  public health nursing practice” (American Association of Colleges of Nursing, 2013) | Provide community assessment in the development of future nursing leaders  Formal/Implicit | *Structure:* community organizations and SON  *Process:* conducting federally mandated community health needs assessments  *Outcome:* integrating health care and public health perspectives on assessment meets both public health and nursing accreditation standards and extends student leadership experiences.  *Implications:* federal mandates for community health needs assessments offer opportunities to advance leadership roles for nursing graduates | #1  Sustaining educational standards and processes - improving academic outcomes |
| Ezeonwu et al., 2014  [37]  USA | Community-based organization/  nursing students, faculty and community partners | Program development & evaluation/ Experiential and non-research evidence  / Level VII | “Community-As-A Partner model” (Anderson & McFarlene, 2008) | Informs students learning through blended learning, for community health nursing services delivery  Formal/Implicit | *Structure:* local community agency and SON  *Process:* community-based education through comprehensive health needs assessments, planning and implementation of disease prevention and health promotion activities for community clients.  *Outcome:* students were challenged to view public health from a broader perspective while analyzing impact of social determinants of health on underserved populations.  *Implications:* 1) extending the environment of teaching and learning beyond the traditional classroom, 2) including atypical settings and online community is a comprehensive approach to teaching community health nursing to RN–BSN students. | #1  Sustaining educational standards and processes - improving  academic outcomes |
| Foster & Barnby, 2018  [68]  USA | Public school / 61 participants, aged 10-13 years/  public school | Descriptive study with pre-test/ post-test/ Level VI | “DRAT! Disaster readiness actions for teens”  (Minnesota Department of Health, 2012) | Collaborative for teaching weather-related disaster preparedness to children  Formal/Implicit | *Structure:* college of nursing, public school, school nurses  *Process:* DRAT program was taught by 4 senior BSNs as a service- learning project and consisted of lecture and interactive group activities.  *Outcome:* 1) participants had a better understanding of disaster preparedness,  2) nursing students thought they made a difference and viewed the program positively.  *Implications:* taught as a service-learning project through pediatric or community health nursing courses, DRAT could increase cost effectiveness in terms of school nurse manpower. | #5  Conceptualizing or implementing innovative academic nursing partnerships |
| Franzese et al., 2020  [62]  USA | Community clubhouse/  8 nursing students | Program evaluation/ Experiential evidence / Level VII | “High-impact education” (Kuh, 2008) | Enhance clinical experiences in mental health nursing  Formal/Explicit | *Structure:* an associate degree nursing program and a community clubhouse  *Process:* nursing students developed wellness-teaching projects based on needs of clubhouse members  *Outcome:* 1) students reported a decrease in stigma in people living with mental illness, 2) clubhouse community highlighted the diversity, knowledge, and preparedness of the nursing students.  *Implications:* Collaboration resolved challenges related to declining clinical sites, stigma associated with mental illness, and fear of working in this field of nursing. | #1 Sustaining educational standards and processes - improving academic outcomes |
| George and DeCristo faro, 2018  [64]  USA | Community rehabilitation facility /  online master’s-level for family NP students | Program evaluation/ Experiential evidence / Level VII | “Health literacy universal precautions toolkit” (AHRQ, 2015) | Enhanced innovative project-based activity for patient education  Formal/Explicit | *Structure:* community rehabilitation facility, community stakeholders  *Process:* students created education pamphlets for teaching health literacy through service learning.  *Outcome:* students met the clinical education and decision support needs of rehabilitation patients while translating academic coursework to support actual community needs.  *Implications:* 1) nursing students can gain experience in working with IP teams.  2) utilizing service learning to teach health literacy as a way to apply content to a real-world environment. | #5  Conceptualizing or implementing innovative academic nursing partnerships |
| Harper et al., 2016  [51]  USA | VA Medical Center/ Nursing faculty, nursing students, and nursing leaders | Program development & evaluation/ Experiential evidence /  Level VII | Veteran Affairs Nursing Academic Partnership (VANAP) Logic Model (Harper, Selleck, Eagerton, & Froelich, 2015) | Improve quality of care for veterans by academic-practice partnerships  Formal/Implicit | *Structure:* SON and VA Medical Center partnership.  *Process:* 3 programs: an undergraduate VA nurse scholars program, a VANAP graduate education program for psychiatric mental health nurse practitioners (NPs), and a Mental Health NP Residency.  *Outcome:* 1) establishment of endowed VA scholarship, 2) job offer to graduates, 3) joint evidence-based practice projects and dissemination through publication and presentation, 4) IP psychiatric rounds for students, 5) development of competencies for residency education, 6) established After-Hours Clinic  *Implications:* 1) top leadership needed from partner organizations, 2) commitment to a shared vision and mutual goals, 3) active engagement from all leaders, 4) data- driven outcomes from each partner, 5) need MOUs to define relationship | #5  Conceptualizing or implementing innovative academic nursing partnerships |
| Iddins et al., 2015  [56]  USA | Free clinic/  M-POWER Ministries /  3 clinicians, a dietitian, RNs, care manager, and a patient assistance program (PAP) coordinator an optometrist | Program evaluation/ Experiential evidence/ Level VII | “Framework for Action on Interprofessional Education & Collaborative Practice” (WHO, 2010) | Interprofessional collaborative practice model for serving the health care needs of an indigent  Formal/Implicit | *Structure:* nurse-driven collaboration between SON, M-POWER Ministries, and University Health System.  *Process:* The PATH Clinic, serving as a setting for learning about team-based care while providing care to medically needy vulnerable populations.  *Outcome:* 1) clinicians and students learned to provide care as an IP team, 2) prevention of costly hospital readmissions, as well as provision of a patient-identified alternative to ED visits for primary care and minor acute illnesses.  *Implications:* 1) refocusing on partnership goal to keep team members aligned and engaged in accomplishing IP objectives, 2) partners need to be thanked often for their service and commitment, 3) quality measurement and process improvement is needed. | #2 Strengthening capacity for collaborative practice and IPE in the community |
| Krol et al., 2016  [76]  USA | Hispanic community/ 130 children and adults | Program development & evaluation/ Experiential evidence/ Level VII | N/A | Integrate community-based learning to address obesity  Formal/Implicit | *Structure:* SON, National Association of Hispanic Nurses (NAHN), and community members  *Process:* Muevete USA™ project: 5 lesson plans on healthy lifestyles for children and families.  *Outcome:* 1) participants increased their score following the lessons, 2) students reported enjoyment of entering in the community, engaging with clients in a new way, feeling empowered to deliver health education, and increased proficiency in working with diverse populations.  *Implications:* When designing a program, it is important to take into account cultural beliefs, and attention to timing. | #4 Enhancing community services and outcomes |
| Krumwiede  et al., 2015  [43]  USA | Hospital and Community-based organization /  15 nursing students partnered with collaborative members | Qualitative case study/ Level VI | “Community-Based Collaborative Action Research (CBCAR) framework” (Pavlish &  Pharris, 2012) | Link service learning and community health needs assessment with public health nursing core competencies  Formal/Explicit | *Structure:* SON, Glen Taylor Nursing Institute for Family and Society (GTNIFS), hospital, and community stakeholders  *Process:* service-learning project *Outcome:* 1) community health needs assessment, 2) students developed skills in core competencies for public health nurses  *Implications:* CBCAR facilitates collaborative partnerships and relationships throughout the research process. | #5  Conceptualizing or implementing innovative academic nursing partnerships |
| Lesley et al., 2013  [73]  USA | Community-based organization / Nursing and physical therapy faculty and APN students | Program evaluation/ Experiential evidence/ Level VII | N/A | Interprofessional partnership to improve physical health status  Formal/Implicit | *Structure:* partnership between a school of health professions and a community mental health services agency  *Process:* a growing physical fitness and health promotion program  *Outcome:* 1) improved health for individuals with serious mental illness and reduced mortality rates associated with unheralded levels of chronic disease, 2) ASLP provided opportunities for practice and research for both APN and physical therapy students.  *Implications:* the public mental health services setting can be a rich training ground for NP students who need to develop expertise in primary care with co-occurring mental and physical conditions | #2 Strengthening capacity for collaborative practice and IPE in the community |
| Levin & Rutkow, 2011  [60]  USA | University setting/  3 health professional schools (Schools of Medicine, Nursing, and Public Health) | Descriptive study/  Level VI | “Service-learning pedagogy”  (Vogel et al. , 2010) | Describes SOURCE’s formation for creating partnerships and services  Formal/Explicit | *Structure:* Student Outreach Resource Center (SOURCE)  *Process:* SOURCE prepared students to collaborate with local partners on a wide range of practice initiatives.  *Outcome:* 1) many health programs were offered, 2) reaching out to faculty and students to develop academic integration opportunities  *Implications:* 1) community engagement experiences are more likely to be valued and utilized if viewed as a part of students’ academic commitments, 2) ongoing challenges were an increased demand for services without a corresponding increase in staffing or financial support. | #2 Strengthening capacity for collaborative practice and IPE in the community |
| McClure et al., 2017  [77]  USA | Home-based care and pediatric asthma specialty clinic (PASC)/  17 patients | Cohort study/ Level IV | N/A | Establish academic practice partnerships to deliver home visit program  Formal/Implicit | *Structure:* SON, nurse students, APN, stakeholders, and PASC.  *Process:* Home visit program (HVP) was conducted by community health nursing students using home environment interventions. *Outcome:* 1) children had fewer asthma related hospitalizations, 2) improved patient and family management of asthma,  3) increased PASC knowledge of asthma, 4) increased student knowledge and skills related to asthma management.  *Implications:* ASLP provided the infrastructure for HVP with the primary goal of extending clinic services and offering a community health clinical practicum. | #4 Enhancing community services and outcomes |
| Merritt & Murphy, 2019  [33]  Guatemala | Free clinic/ 10 Doctor of Nursing Practice (DNP) students | Cohort study/ Level IV | “Kolb’s experiential learning theory” (Kolb, 1984)  “Cultural care theory” (Leininger & McFarland, 2006) | Enhance clinical practice skills and cultural competence via service-learning  Formal/Explicit | Structure: An international service-learning nurse-led clinic, Global Health Immersion course.  *Process:* DNP students and faculty provided primary care in 2 clinics.  *Outcome:* 1) practice competency scores were high overall 2) all students increased their confidence in diagnostic abilities and caring for a culturally diverse population.  *Implications:* An international service learning nurse-led clinic can complement clinical practice hours for NP students in developing diagnostic and cultural competence. | #3 Preparing Nurses of the Future |
| Metcalfe & Sexton, 2014  [42]  USA | Local health department / 87 homeless subjects | Mixed methods survey/ Level VI | “Strategies  for community-academic partnership development” (Baiardi et al., 2010) | Collaborative academic-community partnership to address flu vaccination  Formal/Explicit | *Structure:* a local health department, nonprofit agencies and SON  *Process:* 1) flu vaccination educational social marketing campaign: a service-learning project by nursing students. 2) the local health department developed free flu vouchers. *Outcome:* rate of flu vaccination in homeless persons doubled from previous year.  *Implications:* 1) students engaged in research, learned about population health and worked in collaboration with partners, 2) the local health department reached a vulnerable population. | #4 Enhancing community services and outcomes |
| Miltner et al., 2015  [50]  USA | Veterans Affairs Medical Center/ Nursing students | Program development & evaluation/ Level VII | Veteran Affairs Nursing Academic Partnership (VANAP) Logic Model (Harper, Selleck, Eagerton, & Froelich, 2015) | Describe the evolving partnership for increasing capacity and capability of care providers to Veterans  Formal/Implicit | *Structure:* VA Nursing Academic Partnership (VANAP), the Birmingham Veterans Affairs Medical Center (BVAMC), and SON.  *Process:* exchange of faculty and BVAMC nurse practitioners.  *Outcome:* 1) VANAP undergraduates & graduates employed in VHA, 2) Mental health NP residency, 3) VA Quality Scholars Program Fellows, 4) increased scholarships for nursing students to care for Veterans, 5) cost savings for training and onboarding of new nurses, 6) increased staff development activities, and 7) increased professional presentations and publications.  *Implications:* key to strong partnerships and sustained collaboration is shared goals, mutual trust and respect, the development of formal relationships, and support of senior leadership. | #1 Sustaining educational standards and processes - improving academic outcomes |
| Neubrander et al., 2019  [78]  USA | Free clinic/ 18 Family Nurse Practitioner (FNP) students | Case report/ Experiential & non-research evidence/ Level VII | N/A | Create a free clinic for a rural underserved population and clinical placement for FNP students through a partnership  Formal/Implicit | *Structure:* Golden Leaf Foundation, volunteer-based free clinic, full-time FNP, SON, Cultural competency self-assessment tool.  *Process:* The Increasing Nurse Practitioner in Underserved Territories (INPUT) project: grant funding provided FNP to a clinic and created clinical placements.  *Outcome:* 1) students learned about care for the vulnerable and were encouraged to work in rural communities, 2) increased clinic visits and greater continuity in follow-up care.  *Implications:* ASLPs as models for improving patient health while providing necessary clinical placements for NPs. | #5  Conceptualizing or implementing innovative academic nursing partnerships |
| Petroro et al., 2011  [59]  USA | Local hospital/  5 RNs in MSN program,  19 hospitals | Quality improvement / Experiential evidence/ Level VII | “Council on  linkages between academia and public health  practice” (PHF, 2008) | Describe the collaboration to conduct an active surveillance study  Formal/Implicit | *Structure:* Department of Public Health, SON, and hospitals. *Process:* MSN students in an active surveillance study of vancomycin-resistant enterococcus infections. *Outcome:* 1) students developed skills in 5 of the 8 core competency domains for public health, 2) investigators completed an active surveillance study.  *Implications:* 1) sense of collegiality and trust between students, faculty, and public health professionals leads to further collaborations, 2) challenges: accommodating students’ busy schedules, availability of and access to hospital charts, and missing data. | #5  Conceptualizing or implementing innovative academic nursing partnerships |
| Resha, 2016  [46]  USA | Public and private schools / school nurse, clinical faculty, and student nurse | Experiential and non-research evidence/ Level VII | “The Future of Nursing: Leading Change, Advancing Health” (IOM, 2011) | Enhance school health experience for nursing students to meet the needs of youth  Formal/Explicit | *Structure:* SON, public and private schools *Process:* student nurses participated in community services with school nurses. *Outcome:* 1) school nurses grew professionally as clinical preceptors, 2) students learned about the role of school nurses, developed an understanding of community health, and contributed to population-based health care, 3) nursing faculty lend expertise in research and clinical practice.  *Implications:* evaluation allows for feedback on student performance and helps shape future interactions. | #1 Sustaining educational standards and processes - improving academic outcomes |
| Riedford, 2011  [39]  USA | Community-based organization / nursing students, nursing faculty, community leaders | Qualitative study/  Level VI | “Community Campus Partnerships for Health” (CCPH, 2010) | Engage students in community to provide mental health care  Formal/Explicit | *Structure:* SON, community organizations, a mental health course *Process:* project combined community leaders' ideas for service with innovative suggestions of the students. *Outcome:* students improved understanding of the concepts of citizenship and service to community.  *Implications:* A community project incorporated within a mental health course, where students have exposure to acute and chronic settings, offers a unique understanding of the many facets of mental health across various populations. | #1 Sustaining educational standards and processes - improving academic outcomes |
| Roach & Hooke, 2019  [54]  USA | VA Medical Center/ baccalaureate nursing students, faculty and Veterans Affairs (VA) staff | Program evaluation/ Experiential evidence/ Level VII | “Clinical partnerships” (Didion et al., 2013) | Integrate population health course with veterans’ continuing care through academic-practice partnership  Formal/Implicit | *Structure:* SON, VA, a population health course *Process:* increase student enrollment and support faculty development through VANAP *Outcome:* 1) student groups (inpatient/ outpatient) worked on amputation prevention, 2) continuation of projects by faculty and staff resulted in 47% decrease in vascular-related hospital readmissions.  *Implications:* 1) ASLPs facilitate student learning and improve patient care through faculty involvement in practice innovations, 2) measuring impact on patient outcomes and cost savings underscores the importance of sustaining such partnerships. | #5  Conceptualizing or implementing innovative academic nursing partnerships |
| Sarsfield & Burkhard, 2019  [66]  USA | Community-based organization/ 25 BSN students | Descriptive survey study/ Level VI | “Workplace Health Model” (CDC, 2016) | Examine student perceptions of public health competencies gained through an innovative, nontraditional clinical partnership  Formal/Implicit | *Structure:* non-traditional settings (do not deliver health care), community/ public health nursing course.  *Process:* student volunteer involvement in health promotion activities for employees in organizations. *Outcome:* students rated their experience as influential in the areas of human diversity, health promotion/risk reduction, and communication.  *Implications:* students can achieve community/public health competencies in nontraditional community settings. | #3 Preparing Nurses of the Future |
| Schneider et al., 2018  [32]  Canada & Colombia | Community-based organization/ 10 nursing faculty  9 nursing students | Qualitative study/  Level VI | “Service-learning pedagogy” (Brown and Schmidt, 2016) | Explore the establishment of an ASLP between a nursing program and an isolated community  Formal/Explicit | *Structure:* outskirts community, undergraduate nursing program.  *Process:* semi-structured, in-depth interviews  *Outcome:* 1) learning about social determinants of health, compassion, appreciation for community nursing role, professional growth and development, community engagement and increased access to care.  *Implications:* future studies to evaluate benefits, challenges, and recommendations of service-learning from the unique perspective of the community members. | # 4 Enhancing community services and outcomes |
| Schoon et al., 2012  [61]  USA | Foot care clinic / Nursing faculty, nursing students | Program development & evaluation / Experiential evidence/ Level VII | “A traditional, three-stage, service–learning model—preclinical preparation, immersion, and, post-clinical  reflection” (Hunt, 2007) | Develop a foot care clinic through a long-term academic–community partnership  Formal/Explicit | *Structure:* a local university’s department of nursing  *Process:* during 1^st^ year, students conducted a needs assessment of the homeless shelter population. In the 2^nd^ year, students prepared in holistic health and developed/ piloted the foot care clinic.  *Outcome:* students demonstrated an understanding of homelessness and how to use their citizenship skills for social change within a health care context.  *Implications:* 1) academic support should include a financial commitment for faculty participation and supplies, 2) building a core team of faculty to spearhead the project and provide leadership and mentoring. | #3 Preparing Nurses of the Future |
| Simpson, 2012  [72]  USA | Local hospital / 4 nursing faculty,  200 nursing students,  20 community agencies | Program development & evaluation/ Experiential evidence Level VII | N/A | Offer students to agencies serving vulnerable populations  Formal/Implicit | *Structure:* SON, affiliating hospital, homeless shelters. *Process:* a three-year assignment to agencies serving vulnerable populations. *Outcome:* 1) students had a better understanding of the community, 2) faculty enjoyed community work and requested permanent assignment to courses  *Implications:* 1) faculty had to be flexible and creative when scheduling student rotations, 2) students were exposed to a variety of community programs and populations. | #1 Sustaining educational standards and processes - improving academic outcomes |
| Strickland et al., 2014  [34]  USA | Community-based organization / American Indian tribes, 60 undergraduate, 13 graduate students (11 DNP and 2 MN, and 3 faculty members | Qualitative study using community based participatory research/  Level VI | “Transcultural Nursing Concepts” (Leininger et al., 2002) | Implement education programs and research in tribal communities    Over 3 years, students and faculty worked with 2 tribal communities to design research and implement education programs.  Formal/Explicit | *Structure:* 2 tribal communities, SON, National Institute of Nursing Research grant, community agency partners, tribal clinical preceptors *Process:* employed tribes to serve as the Community Research Associate and provide them access to university resources. In turn, they mentored and engaged students in program planning and research to meet tribal health concerns. *Outcome:* 1) understanding of the community health nurse role and ability to translate research evidence into practice,2) understanding of Pacific Northwest American Indian cultural values, beliefs, patterns of communication and health concerns.  *Implications:* 1) model provided students and tribal representatives an opportunity to work collaboratively in program planning and research proposal development, 2) flexibility, attention to communication, and collaborative structures contributed to success, 3) combining teaching, research, and practice in partnership building is of value to nurse educators seeking creative ways to build community partnerships. | #5  Conceptualizing or implementing innovative academic nursing partnerships |
| Sullivan-Marx et al., 2010  [69]  USA | Community-based organizations/ 381 members (mostly women and African Americans) | Case study/ Experiential and non-research evidence/ Level VII | “Program of All-Inclusive Care for Elders (PACE) model” (Mukamel et al., 2007) | Establish community-based long-term care program to high-risk older adults  Formal / Implicit | *Structure:* SON, chief nursing officers. *Process:* the Living Independently For Elders (LIFE) 10-year program; collaborative partnership between LIFE members/families and SON to provide community‐based long‐term care.  *Outcome:* 1) higher level of engagement with policy makers at the state and national level, 2) greater understanding and feasibility of nursing science and care to other disciplines  *Implications:* The program can be used globally and strengthen service partnerships | #5  Conceptualizing or implementing innovative academic nursing partnerships |
| Sutter-Barrett et al., 2019  [70]  USA | Public school /  over 1000 public school students | Program development & evaluation/ Experiential evidence/  Level VII | “Bridge Care Model”  (Sutter-Barrett et al, 2015) | Implement Bridge Care Model through an innovative partnership  Formal/Implicit | *Structure:* public school, academic nurse-managed clinic network, SON-based staff  *Process:* “Bridge Care Model” provide access to medical navigation support for uninsured families and vulnerable students for school entry.  *Outcome:* improving access to health service, reducing delayed enrollment, and offering health professions students an opportunity to provide interdisciplinary direct and referral health services  *Implications:* 1) academic institutions working with communities offer substantial resources which can be shaped into innovative solutions, 2) program increased service capacity and raised nursing students’ awareness. | #5  Conceptualizing or implementing innovative academic nursing partnerships |
| Swenty et al., 2016  [57]  USA | VA Medical Center/ Graduate nurse practitioner, social work, occupational therapy students and undergraduate nursing, respiratory therapy, and nutrition students | Program development & evaluation/ Experiential and non-research evidence/ Level VII | “Clinical interprofessional model” by College of Nursing and Health Professions (CNHP) (Dobalian et al., 2014) | Implement an IP model to optimize veteran health outcomes  Formal/Implicit | *Structure:* SON and Veterans (VA) interprofessional Patient Aligned Care Teams (PACT). *Process:* Partnering IP student teams with VA health professionals (including at home follow-up of VA patients).  *Outcome:* nursing students had a better understanding of the veteran population.  *Implications:* interprofessional collaborative practice (IPCP) using PACT team principles ultimately promotes the students' ability to link theory content to patient care delivery. “The key to a successful academic-VA relationship is the development of mutual goals, trust, and respect” (Miltner et al., 2015). | #2 Strengthening capacity for collaborative practice and IPE in the community |
| Testut, 2019  [65]  USA | Hospital and homeless shelters/ Students from the RN-to-BSN and RN-to-BSN-to-MSN programs and other programs, including occupational therapy and social work | Program development & evaluation/ Experiential evidence  Level VII | “The Camden Model” (Camden Coalition of Healthcare Providers, 2018) | Establish academic partnership program “iCAN” to navigate services for vulnerable clients  Formal/Implicit | *Structure:* college of nursing, law enforcement, affiliated hospitals and representatives from communities and homeless shelters. *Process:* students participated in the Interprofessional Community-Academic Navigation program (iCAN) to fulfill clinical or field experience hours. *Outcome:* Increased awareness into social determinants of health and improved client navigation to community services.  *Implications:* 1) The iCAN program can adapt to new challenges in healthcare, higher education, and population diversity 2) Development of an IP community-academic partnership is in line with the population health concept. | #2 Strengthening capacity for collaborative practice and IPE in the community |
| VanGraafeiland et al., 2019  [58]  USA | University/ 20 pre-licensure MSN (Entry into Nursing) students | QI study/ Level VII | “Health Professional Education in Patient Safety Survey and the Systems Thinking Scale” (Ginsburg et al., 2012) | Develop an academic-clinical service partnership to  to advance patient safety competence and leadership in students  Formal/Implicit | *Structure:* the Helene Fuld Leadership Program *Process:* students are assigned to mentored, quality improvement (QI) projects over 2 semesters. *Outcome:* improved student knowledge of patient safety principles and promoted competence in patient safety with the development of future patient safety and QI nurse leaders.  *Implications:* a mutually beneficial program that provided mentored learning opportunities for students and concrete support for clinicians and faculty involved in QI projects. | #3 Preparing Nurses of the Future |
| Voss et al., 2015  [15]  USA | Community-based organization/  3 community partner representatives,  2 nursing faculty, and 6 UG nursing students | Descriptive study/  Level VI | N/A | Implement service–learning projects in community through a collaborative community–academic partnership  Formal/Explicit | *Structure:* SON, community partners Process: 2 phases comprised the framework development process; -Phase I community partners faculty collaborated to identify key elements for reciprocal service–learning.  -Phase II, students piloted the draft framework during population-based care course. *Outcome:* 4 major themes emerged; access, feasibility, data collection, and consistency.  *Implications:* 1) The service–learning framework is the product of a Community Academic Partnership (CAP) to support collaborative project planning and reciprocity, 2) Nursing faculty need to develop a plan for measuring the benefit to those receiving the service. | #5 Advocating for innovative academic nursing partnerships |
| Wros et al., 2015  [36]  USA | Community service organizations and primary care clinics/  2 case studies and IP student teams, including nursing, medicine, pharmacy, and dentistry | Case study/ Experiential evidence/  Level VII | “Triple Aim” (Berwick et al. 2008)  and  “Social determinants of health framework” (WHO, 2013) | Implement academic-practice model (I-CAN) to improve the health of underserved neighborhoods  Formal/Implicit | *Structure:* community service organizations, primary care clinics, the Interprofessional Care Access Network (I-CAN). *Process:* I-CAN project: IP student teams collaborated with community to address social determinants of health for achieving Triple Aim goals. *Outcome:* 2 case studies demonstrated that I-CAN project 1) provided students with authentic learning experiences, 2) enhanced health navigation by developing relationships between clients and primary care providers in preventive health care, and reduced or eliminated inappropriate and costly use of services.  *Implications:* Education and practice models such as I-CAN can contribute to development of an inclusive and effective system of health care in the US | #5  Conceptualizing or implementing innovative academic nursing partnerships |
| Wu et al., 2013  [63]  USA | Local public schools/  36 BSN students,  1,800 middle- and high school students and their families | Mixed methods study/  Level VI | “ LIVE  (learning, inviting, valuing, and engaging) conceptual framework” (Bernick & Clark, 2008) | Collaborative partnership  for increasing awareness of colorectal cancer (CRC) screening  Formal/Explicit | *Structure:* BSN students and local public schools. *Process:* nursing students prepared school aged students as health ambassadors to deliver messages about colorectal cancer screenings for significant others (age 50 and older). *Outcome:* considering to specialize in community health nursing correlated moderately with both gaining knowledge about community health and developing skills working with minority populations.  *Implications:* 1) program provides unique service-learning experience while improving knowledge and clinical skills, 2) integrate as a regular component of the community nursing course, 3) outcomes can lead to development of evidence-based practice for community health nurses. | #4 Enhancing community services and outcomes |

* Adapted *Rating System for the Hierarchy of Evidence* (Fineout-Overholt et al, 2010; Dang & Dearholt, 2017)

**According to Donabedian’s Quality of Care (1988) *ASLNP Structure*: physical and organizational components of the partnership *ASLNP Process*: learning methods and services provided to recipients *ASLNP Outcomes:* results of learning methodology and services rendered

§ Academic-Service Learning Nursing Partnership Themes:

1. Sustaining educational standards and processes - improving academic outcomes

2. Strengthening capacity for collaborative practice and IPE in the community

1. Preparing Nurses of the Future
2. Enhancing community services and outcomes
3. Conceptualizing or implementing innovative academic nursing partnerships
